# Comparison of the Ability of Static and Dynamic Balance Tests to Determine the Risk of Falls among Older Community-Dwelling Individuals

**DOI:** 10.3390/jfmk8020043

**Published:** 2023-04-07

**Authors:** Puttipong Poncumhak, Arunrat Srithawong, Winut Duangsanjun, Patchareeya Amput

**Affiliations:** 1 Department of Physical Therapy, School of Allied Health Sciences, University of Phayao, Phayao 56000, Thailand; 2 Unit of Excellence of Human Performance and Rehabilitations, University of Phayao, Phayao 56000, Thailand; 3 Adult and Gerontological Nursing, School of Nursing, University of Phayao, Phayao 56000, Thailand

**Keywords:** falls, prediction, balance impairment, diagnostic accuracy, screening

## Abstract

This study aimed to examine the validity of balance tests and compare their diagnostic accuracy to determine the risk of falls among older community-dwelling individuals. Eighty-five older participants were assessed based on their demographics and fall data. They were then assessed for the ability to perform balance measures, including five times sit-to-stand tests (FTSSTs), timed up and go tests (TUGs), three times stand and walk tests (TTSWs), functional reach tests (FRTs), and single-leg stance tests (SLSs). The correlation between fall data and balance measures was found to be significant for all parameters (*p* < 0.05). The TTSW showed the highest level of ability to indicate the risk of falls among older community-dwelling adults with sensitivity = 92.68%, specificity = 84.09%, and AUC = 0.931 (95%CI = 0.860 to 1.000). These findings confirm the benefits of the practical functional balance measures to determine the risk of falls among older community-dwelling individuals.

## 1. Introduction

Balance impairment among older individuals contributes significantly to multiple body functions in terms of mobility, exercise capacity, functioning, and health, which consequently has negative impacts on the ability to conduct daily activities, thus enhancing the risk of falls and fractures [1,2]. Moreover, falls among older adults are a major contributor to loss of independence, hospitalisation from trauma, and injury-related fractures, as well as decreases in quality of life, associated health care costs, and death rate [3]. Therefore, the balance impairment factor is commonly used to assess the risk of falling in older people. However, although balance impairment in older individuals is well-known as an important predictor of falls, many studies have been limited in recommending specific clinical assessment scales of balance [4,5,6]. Therefore, it is strongly recommended to explore a screening test that aids in early detection and periodic follow-up so that falls can be prevented, and effective management and treatment can be carried out.

The balance measurement tools that are currently used in clinical settings are a means of quantifying the working capacity of the sum of the components that enable postural stability [7]. The goal of screening is to identify individuals who are at an increased risk for falling and should thus receive further assessment [7]. Therefore, screening should utilise practical and easy-to-administer measurement tools that are able to identify impairments associated with future fall risk. Many studies have recommended functional field-based balance assessments that roughly indicate levels of functioning among individuals and are simple, feasible, practical tools that can be carried out in almost any setting. They are easily administered to larger populations, and the outcomes could suggest further assessments for the systems with disorders [2,8,9]. Therefore, functional assessments are commonly applied as screening and monitoring tools [2].

There are a variety of clinical assessment scales of balance that have been proposed for use to assess the risk of falling. Based on the easy-to-administer measurement which can be applied to a large population in the community, the researchers are interested in functional balance tests that include dynamic and static balance tests, comprising five times sit-to-stand tests (FTSSTs), timed up and go tests (TUGs), three times stand and walk tests (TTSWs), functional reach tests (FRTs), and single-leg stance tests (SLSs). The FTSST has excellent test–retest reliability and inter-rater reliability (intraclass correlation coefficients (ICCs) = 0.99 and 0.99, respectively) [10] to reflect functional lower limb muscle strength and dynamic balance control [10,11]. The TUG is a test with excellent inter-rater reliability (ICCs = 0.97 to 0.99) [12]. Thus, outcomes of the test are commonly used to reflect dynamic balance control and risk of fall of older individuals [13]. In addition, the TTSW we previously developed has excellent reliability (ICCs = 0.991; 95% CI = 0.984 to 0.996) [14]. The outcome of the test is used to indicate the risk of falling and frailty in older individuals [15,16]. Other balance tests, including FRT and SLS, have been reported to be useful for detecting balance impairment in the older individuals [17,18]. The tests are portable, inexpensive, reliable, precise, and have reasonable clinical application [17]. However, no comparative reporting of the fall risk indications of these tests has yet been found. Therefore, this study aimed to report the validity of the tests and compare the ability of these tests to determine the risk of falls among older community-dwelling individuals. 

## 2. Methods

### 2.1. Study Design and Participants 

This cross-sectional study involved 85 participants who were older community-dwelling adults aged 60 years and above. The inclusion criteria were a body mass index between 18.5 and 22.9 kg/m^2^, the ability to stand up independently, the ability to walk without external assistance, with or without a walking device, and the ability to understand the commands used in this study. The exclusion criteria were any signs and symptoms that might affect participation in the study, such as uncontrolled medical conditions (hypertension or heart disease, etc.) or pain in the musculoskeletal system that might affect the outcomes of the study, with a pain score of more than 5 out of 10 on a visual analogue scale. 

The minimum sample size was estimated from a primary objective (diagnostic accuracy), and the data from a pilot study (*n* = 30) provided a sensitivity range of 68–85%. We set the power of the test at 90%, a *p*-value of 0.05, and the lowest sensitivity value from a pilot study of 0.68. The findings indicated that the study required at least 85 participants. All participants signed written informed consent forms prior to participation in the study. The research protocols were approved by the Institutional Ethics Committee for Human Research (EC No. 1.2/013/65).

### 2.2. Research Protocols

Eligible participants were interviewed and assessed for their demographics and fall data. A fall in this study is defined as any unintentional event that results in a person coming to rest on the ground, neither as a result of a major intrinsic event such as stroke or syncope, nor an extrinsic force/overwhelming hazard such as forcefully being pushed down or having a road traffic accident [19,20]. The fall data were recorded for the number of falls in the previous six months with their related events, such as the cause of falls, time, location, activity during falls, and experience of falls-related injuries [19,21]. Participants were then divided into non-faller and faller (who had experienced one or more fall events) groups. They were then randomly sequenced to assess their ability to perform functional measures to reflect the balance ability of older individuals, including FTSST, TUG, TTSW, OLS, and FRT. Details of the tests are as follows.

1. Five Times Sit-to-Stand Test: The participants sat on a chair with their back upright and hip flexion at 90 degrees. Their feet were placed flat on the floor behind their knees, and their arms were crossed over their chest. Subsequently, the participants completed 5 chair-rise cycles at the fastest and safest speed possible, without using their arms. The test was repeated over 3 trials; average data were recorded in seconds [22].
2. Timed Up and Go test: The participants stood up from a standard armrest chair (with a seat height of 43–46 cm), walked around a traffic cone that was placed 3 m from the front edge of the chair, and then returned to sit down on the chair. The time from the command “Go” until the participant’s back touched the backrest of the chair was recorded in seconds. The average time over the 3 trials was reported [22,23,24].
3. Three Times Stand and Walk test: The TTSW test was previously developed for assessing muscle strength, gait, and balance ability among older community-dwelling individuals [15]. The participants were required to sit on a standard armless chair with their backs upright and a hip flexion of 90 degrees. Their feet were placed flat on the floor approximately 10 cm behind the knees, and their arms were left at their sides. Afterward, the participants were instructed to stand up repeatedly with their hips and knees in full extension, sit down three times, then walk around a traffic cone placed 3 m from the front edge of the chair, and return to sit down on the chair at the fastest, safest speed [15]. The average findings of the three trials were used for data analysis.
4. The Functional Reach Test: This test was performed using a levelled yardstick attached to the wall at the height of the participant’s right acromion process. The examiner recorded the initial and end reach positions. In the starting position, participants stood comfortably and placed their right arm parallel to the yardstick without touching the wall, and the examiner recorded the initial positions at third metacarpal along the yardstick. Participants then reached as far forward as they could without losing their balance (end position), and an examiner recorded their end positions [17,25].
5. The Single-Leg Stance test: The participants were asked to stand on one lower limb of their choice and cross their arms over their chests, with the other limb raised off the floor without touching anything else. The investigator recorded the length of time that participants were able to stand on one limb. Time commenced when the participant raised a foot off the floor and ended when the participant either (1) uncrossed their arms, (2) dropped their raised foot to touch the floor, (3) moved their weight-bearing foot, or (4) a maximum of 60 s had elapsed. The procedure was repeated 3 times, and the average of the 3 trials was recorded [18,26].

During the tests, the participants wore a lightweight safety belt, with a physiotherapist walking or being alongside the participants throughout the tests to ensure the participants’ safety and improve the accuracy of the outcomes. In addition, the participant took no more than 5 min to complete each test, including the rest period of 30 s between trials of each test. 

### 2.3. Statistical Analysis

Descriptive statistics were used to explain the personal data and findings of the study. An independent sample *t*-test was applied to indicate the discriminative ability of the balance measures for faller and non-faller participants. The point biserial correlation coefficient (rpb) was employed to quantify the levels of correlation between the dichotomous outcome of fall data (yes/no) and the outcome of the balance measures. Spearman’s rank correlation coefficient (rho) and Pearson correlation coefficient (r) were employed to quantify the levels of correlation between the outcomes of the balance measures. The receiver operating characteristic (ROC) curves were further utilised to verify the diagnostic accuracy of the balance measures to indicate falls by exploring the optimal cut-off score, sensitivity, specificity, and area under the ROC curve (AUC). The level of statistical significance was set at *p* < 0.05. 

## 3. Results 

Eighty-five eligible participants were recruited in the current study, with an average age of 70 years. There was no significant difference in the average age and BMI between fallers and non-fallers (*p* > 0.05, Table 1). The results showed a significant difference in the outcomes of FTSST, TUG, TTSW, FRT, and SLS between the fallers and non-fallers (*p* < 0.001, Table 1). 

Of all participants, 41 had experienced at least one fall. The total number of falls was 94, ranging from 1 to 5 times (Table 2). Table 2 presents the circumstances of falls wherein most of the falls occurred while walking (50%) in the community or workplace (40.43%) during the daytime (58.51%); falls reported were most often caused by loss of balance (34.04%). Although most participants reported no serious consequences caused by falling (65.96%), of the five times reported to have required medical attention, two participants had fractures caused by falls. 

The correlation between fall data (number of falls) and balance measures was found to be significant for all parameters (*p* < 0.05, Table 3). Of all correlation levels between fall data and balance measures, the TTSW test was found to have the highest correlation with fall data (rho = 0.732, *p* < 0.001, Table 3). Meanwhile, assessment of the correlation level between balance measures found that the FTSST and TUG tests had the highest level of correlation (rho = 0.778, *p* < 0.001, Table 3). Table 4 presents the diagnostic accuracy of five balance measures to determine falls among older individuals. Based on sensitivity, specificity, and AUC, the TTSW showed the highest level of ability to indicate the risk of falls among older community-dwelling adults, with sensitivity = 92.68%, specificity = 84.09%, and AUC = 0.931 (95%CI = 0.860 to 1.000) (Table 4). 

## 4. Discussion 

Based on the easy-to-administer measurement and the ability to be applied to a large population in the community, this study confirmed the use of these balance measures as a practical screening tool for older community-dwelling individuals. Use in this population can be confirmed based on the correlation levels and values related to diagnostic accuracy variables, including sensitivity, specificity, and AUC. The important finding was that the TTSW had the highest level of correlation with fall data (Table 3). It was also found to have the highest level of predictive ability for fall risk, followed by SLS, FTSST, TUG, and FRT, respectively (Table 4).

Postural balance control has been defined as the ability of a participant to maintain the centre of pressure (CoP) within the base of support to maintain balance, orientation, and prevent falling [27]. In daily life, the maintenance of postural control is essential under both static and dynamic conditions [28]. The static condition is referred to as balance under unperturbed environments such as quiet standing, while the dynamic condition is connected to the ability of the participant to react efficiently to displacements or external mechanical stimuli on the base of support [28,29]. Although dynamic postural control plays a critical role because individuals are subjected to different threats during their daily activities and dynamic states, static postural control is also important and necessary to maintain the CoP during sitting or standing, especially for older individuals [30]. The present study found a strong correlation between fall history and the SLS as a static balance measure (rho = 0.703, *p* < 0.001, Table 3). Hence, static postural balance control is as necessary as dynamic control. 

For dynamic balance measures, the TTSW showed the highest correlation level and diagnostic accuracy (Table 3). The TTSW was previously developed based on the combination of muscle strength, walking, and balance abilities in the lower extremities, which are important factors that increase the risk of falling among older individuals. Previously, the test had a significant correlation with standard tests, including the FTSST (r = 0.648, *p* < 0.001) and the TUG (r = 0.673, *p* < 0.100) [14]. Moreover, the outcome of the TTSW is used to indicate the risk of falling in older individuals [15]. Therefore, it is possible that the characteristics of the test contributed to finding a higher level of correlation and ability to indicate fall risk than other dynamic balance measures. 

This study was conducted on only one static balance measure, the SLS. The test requires the ability to stand on one leg and keep the centre of mass above the centre of pressure, as reflected in the variability of ground reaction forces (GRFs) [31]. Although the SLS seems particularly easy to perform with the eyes open, some older individuals with conditions may find it difficult to perform. Therefore, this study showed that SLS had a high level of correlation with the history of falls (Table 3) and had the highest level of ability to indicate fall risk after TTSW (Table 4). However, although the test was completed after 60 s had elapsed [26], some participants were still able to continue the test beyond this time. Therefore, the outcomes of the test may face ceiling effects in high-functioning older people. The current findings further suggest the use of the TTSW to reflect and determine the risk of falls among older individuals. 

In general, an AUC of 0.5 suggests no discrimination (i.e., ability to diagnose patients with and without the disease or condition based on the test), 0.7 to 0.8 is considered acceptable, 0.8 to 0.9 is considered excellent, and an AUC greater than 0.9 is considered outstanding [32]. The present study provides the optimal cut-off score of the functional balance tests with acceptable-to-outstanding discriminative ability (Table 4). Among all the tests, it was found that the TTSW had the highest predictive value, followed by the SLS, both of which had outstanding predictive abilities. The TTSW is very challenging and demanding for muscles in the lower extremities, and for assessing balance control and walking ability [16]. While the SLS is a test that reduces the base of support and appears to be a simple test, it is actually a very difficult task for older individuals who need to maintain posture in a stationary position on the narrow base of support for as long as they can. These findings suggest a clinical benefit of the SLS as a simple test with practical ability to screen risk of falls in older individuals. Although the TTSW had the highest AUC, it may require more equipment and therefore may be suitable for the older individuals with high physical abilities. 

There were some limitations in this study. Firstly, the number of participants in each group and the number of female participants outnumbered male participants, which may have affected the results. However, no significant difference was observed between the number of female fallers and non-fallers. Second, the balance measures compared in this study were obtained on the basis of a simplified review of the literature, which may not have covered other interesting tests. Therefore, further studies may need to include other tests and a more precise selection process for the various tests.

In conclusion, the findings in this study confirm the benefits of practical functional balance measures to determine fall risk among older community-dwelling individuals. On the basis of all the balance measures in this study, the TTSW is suggested as the best screening test. Such measures may be incorporated to promote standard and effective community-based rehabilitation and home healthcare services. 

## Figures and Tables

**Table 1:** The demographics of the participants and the outcome of balance measures.

Variables	Total (*n* = 85)	Faller (*n* = 41)	Non-Faller (*n* = 44)	*p*-Value
Age: years ^a^	70.24 ± 8.80	70.78 ± 9.78	69.73 ± 7.85	0.584
Gender: *n* of female (%)	56 (65.88)	29 (70.73)	27 (61.36)	0.493
BMI: kg/m^2 a^	23.30 ± 4.34	22.95 ± 4.44	23.62 ± 4.27	0.480
Five times sit-to-stand test: s ^a^	12.29 ± 3.82	14.82 ± 3.68	9.92 ± 2.00	<0.001
Timed up and go test: s ^a^	10.95 ± 3.42	13.00 ± 3.69	9.05 ± 1.57	<0.001
Three times stand and walk test: s ^a^	12.82 ± 2.80	14.97 ± 2.66	10.86 ± 0.76	<0.001
Functional reach: cm ^a^	23.04 ± 8.38	18.98 ± 7.34	26.82 ± 7.52	<0.001
Single-leg stance test: s ^a^	27.79 ± 16.56	15.83 ± 11.74	38.94 ± 11.98	<0.001

^a^ The values are expressed by mean ± SD.

**Table 2:** Fall data: causes, times, locations, circumstances, and fall-related injuries as perceived by the participants.

Falls Information		Frequency (%)
1. Number of falls: previous 6 months ^a^	- 1 time	9 (21.95)
	- ≥2 times	32 (78.05)
2. Cause of falls ^b^	- Loss of balance	32 (34.04)
	- Trip	21 (22.34)
	- Slip	24 (25.53)
	- Leg muscle weakness	17 (18.09)
3. Time of falls ^b^	- Day time	55 (58.51)
	- Evening and night-time	49 (41.49)
4. Location of fall ^b^	- Inside house	34 (36.17)
	- Outside house	22 (23.40)
	- Community or workplace	38 (40.43)
5. Activity during falls ^b^	- Walking	47 (50.00)
	- Changing posture	43 (45.74)
	- Standing	4 (4.26)
6. Experience falls-related injuries ^b^	- No injury	62 (65.96)
	- Injury without medical attention requirement	27 (28.72)
	- Injury with medical attention requirement	5 (5.32)

Note: The fall data were recorded for the number of falls in the previous six months. ^a^ The number of fallers was 41 participants. ^b^ The total number of falls was 94.

**Table 3:** Correlation between outcome of balance measures and falls history.

Variables	SLS		FRT		TTSW		TUG		FTSST	
	rho	*p*-Value	rho	*p*-Value	rho	*p*-Value	rho	*p*-Value	rho	*p*-Value
**Number of Falls**	−0.70 *	<*0.001*	−0.470 **	<*0.001*	0.732 **	<*0.001*	0.581 **	<*0.001*	0.645 **	<*0.001*
**FTSST**	−0.540 **	<*0.001*	−0.454 **	<*0.001*	0.556 **	<*0.001*	0.778 **	<*0.001*		
**TUG**	−0.496 **	<*0.001*	−0.458 **	<*0.001*	0.451 **	<*0.001*				
**TTSW**	−0.615 **	<*0.001*	−0.260 *	*0.016*						
**FRT**	0.421 **	<*0.001*								

* Significant at *p*-value < 0.05; ** Significant at *p*-value < 0.001. FTSST, five times sit-to-stand; FRT, functional reach test; SLS, single-leg stance test; TTSW, three times stand and walk test; TUG, timed up and go test.

**Table 4:** The cut-off score of the balance ability measures to determine falls in older individuals.

Balance Measures	Cut-Off	Sensitivity (%)	Specificity (%)	Correctly Classify (%)	AUC	(95%CI)
Five times sit-to-stand test	≥10.72 s	87.80	61.36	84.71	0.887	0.817–0.957
Timed up and go test	≥9.44 s	85.37	65.91	74.12	0.827	0.739–0.915
Three times stand and walk test	≥11.8 s	92.68	84.09	84.09	0.931	0.860–1.000
Functional reach	<22.67 cm	77.27	63.41	70.59	0.760	0.660–0.860
Single-leg stance test	<23.00 s	90.91	78.05	84.71	0.909	0.850–0.969

AUC, area under receiver operating characteristic (ROC) curve; CI, confidence interval.

## Data Availability

Data are available from the corresponding author upon reasonable request.
